# Advances in 3D bioprinting for urethral tissue reconstruction

**DOI:** 10.1016/j.tibtech.2023.10.009

**Published:** 2023-12-05

**Authors:** Daniel Booth, Ronak Afshari, Mahsa Ghovvati, Kaavian Shariati, Renea Sturm, Nasim Annabi

**Affiliations:** 1 Department of Chemical and Biomolecular Engineering, University of California, Los Angeles, Los Angeles, CA 90095, USA; 2 David Geffen School of Medicine, University of California, Los Angeles, Los Angeles, CA 90095, USA; 3 Department of Urology, David Geffen School of Medicine, University of California, Los Angeles, Los Angeles, CA 90095, USA; 4 Department of Bioengineering, University of California, Los Angeles, Los Angeles, CA 90095, USA; 5 https://www.uclahealth.org/providers/renea-sturm; 6 http://www.annabilab.ucla.edu/

## Abstract

Urethral conditions affect children and adults, increasing the risk of urinary tract infections, voiding and sexual dysfunction, and renal failure. Current tissue replacements differ from healthy urethral tissues in structural and mechanical characteristics, causing high risk of postoperative complications. 3D bioprinting can overcome these limitations through the creation of complex, layered architectures using materials with location-specific biomechanical properties. This review highlights prior research and describes the potential for these emerging technologies to address ongoing challenges in urethral tissue engineering, including biomechanical and structural mismatch, lack of individualized repair solutions, and inadequate wound healing and vascularization. In the future, the integration of 3D bioprinting technology with advanced biomaterials, computational modeling, and 3D imaging could transform personalized urethral surgical procedures.

## Clinical need for engineering tissue constructs to treat urethral diseases

A wide range of malignant, traumatic, infectious, or developmental conditions can result in abnormalities of the urethra, with significant effects on quality of life and healthcare costs due to painful or obstructed urination, urinary tract infections/urosepsis, sexual dysfunction, urinary retention, and renal failure [1]. Two of the most common etiologies are hypospadias and **urethral stricture disease** (see Glossary). A surgical procedure that repairs or replaces a section of the urethra, termed as **urethroplasty**, is often required in affected individuals.

Hypospadias is a common congenital condition, affecting approximately one in 250 boys [2]. In adults, urethral stricture disease affects about one in 150 men [3]. Common etiologies are idiopathic, traumatic, iatrogenic, and inflammatory or infectious conditions [4]. In both conditions, affected regions, urethral length, and tissue properties vary widely and require personalized repair approaches. Current tissue sources used for complex or redo urethroplasties include preputial or **buccal** grafts [5], which are limited by tissue availability and donor site morbidity [6]. There is no current FDA-approved product designed for urethral replacement, limiting options for surgeons in need of healthy urethral tissue alternatives. Extensive or complex urethral repairs are particularly high risk, with 49–68% intermediate-term risk of complications reported for proximal hypospadias repairs in recent series [7].

Common urethral complications include **fistula**, **diverticulum**, or stricture, highlighting the need for an improved surgical repair alternative that supports lifelong unobstructed cyclic voiding [8,9]. In individuals with urethral conditions corrected by urethroplasty, key factors leading to these postoperative complications include structural and mechanical mismatch between the native and reconstructed urethra, lack of individualized repair solutions, and inadequate wound healing and vascularization.

**Figure 1.**
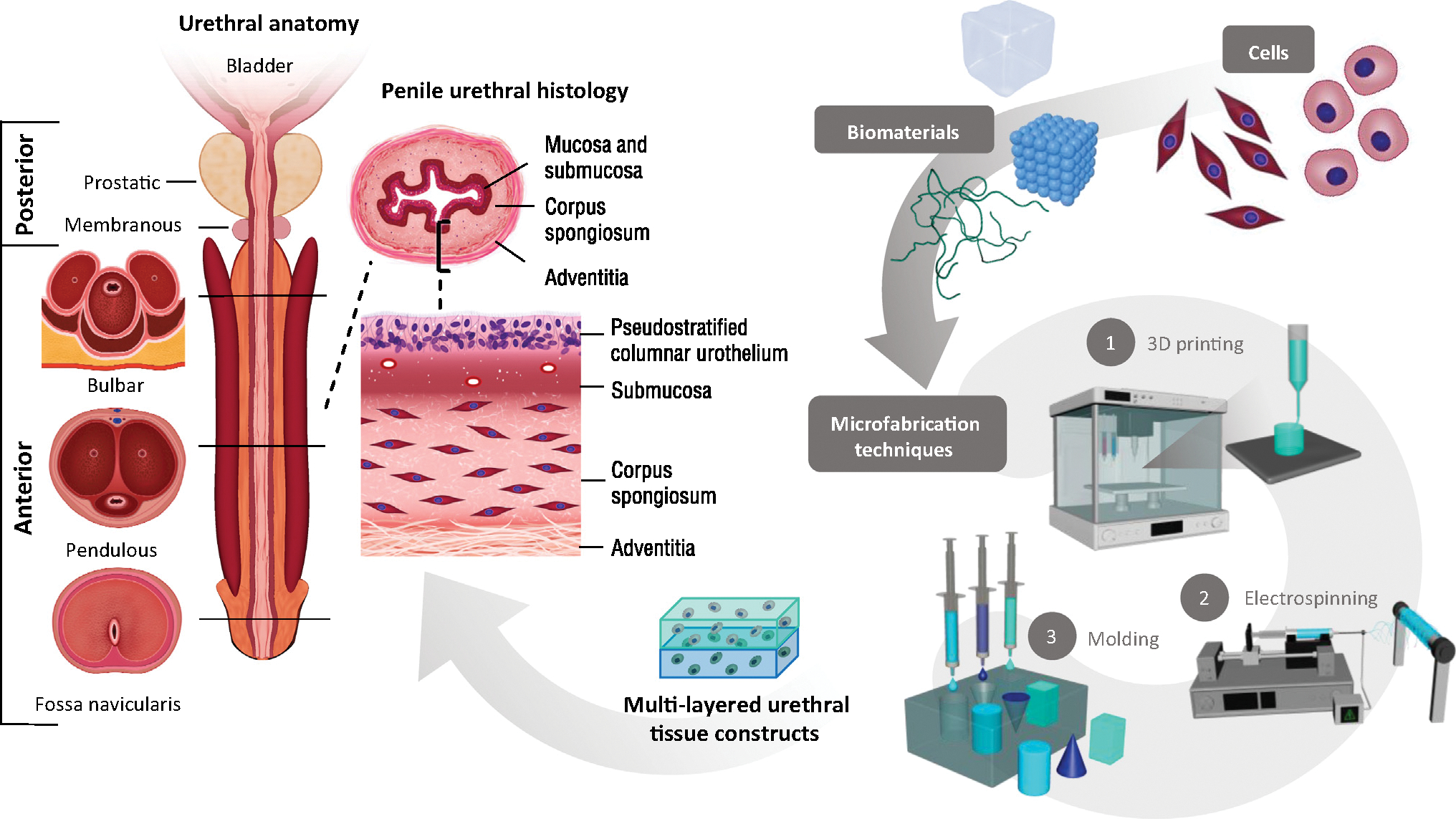
Anatomic, structural, and pathologic features of the male urethra. These are critical features to guide the application of novel biomaterials, cell seeding, and microfabrication techniques in the development of personalized multilayered tissue-engineered constructs.

As illustrated in the cross-sectional images in Figure 1, anterior urethral position, structure, supporting tissue, and histology are temporally distinct along its length [10]. Grafts used in urethroplasty fail to recreate this multilayered support [11]. Specifically, buccal or preputial grafts aim to preserve only an epithelial layer and submucosa, while omitting outer layers of smooth muscle and **corpus spongiosum** that provide radial support to the native urethra [12]. Furthermore, current graft sources also have distinct epithelium that differs from urothelium which is organized in a location-dependent manner (e.g., prostatic: transitional, membranous and penile: pseudostratified and stratified columnar, and fossa navicularis: nonkeratinized stratified squamous urothelium) [10]. Buccal grafts, however, are lined by nonkeratinized stratified squamous [12] and foreskin by keratinized stratified squamous epithelium [13]. These differences affect multiple aspects of urethral function, including resistance to urinary metabolites and prevention of urine extravasation [14]. Put together, structural differences in grafts versus native tissue highlight the need for an engineering approach that can create modifiable multilayered structural support and enhance site-dependent epithelialization.

Beyond their structure, another area in which current autografts differ significantly from the native urethra is their tensile properties. For example, the two of the most common alternative tissue sources for urethroplasty have tensile moduli ≥100× the urethra. Human buccal mucosa has a mean tensile modulus of 8.3 ± 5.8 MPa [15], while that of **prepuce** is 2.84 ± 0.25MPa [16]. This contrasts with the softer, elastic tissue of the male anterior urethra, which has a mean tensile modulus of 0.034 ± 0.01 MPa [17]. These unique urethral tensile and structural properties require a modern materials approach that facilitates the selection and fabrication of tunable materials to minimize localized wound tension and enhance urination and **tumescence** after anastomosis of the material to urethral tissue.

Finally, wound healing is affected by microscopic and macroscopic factors, including vascularization and local immunomodulation. For example, angiogenic activity within varied grafts is critical for viability following a urethroplasty and varies between tissue types [18]. Graft vascularization and tissue regeneration have additionally been enhanced through the addition of select biochemical cues [19] and progenitor cell seeding [20,21]. Put together, an engineered scaffold that provides biochemical and structural cues to enhance early angiogenesis is needed to ensure graft vascularization and scaffold replacement with regenerated urethral tissue.

A promising engineering strategy that is poised to meet each of these key ongoing challenges to a successful urethroplasty is 3D bioprinting. Biomaterials with tunable mechanical properties can be printed to create individualized, multilayered constructs with cell deposition and varied biochemical cues by temporal position. Although prior review articles have discussed lower urinary tract tissue engineering [21–23], and the application of 3D bioprinting in urology [24–27], the present review uniquely describes the cutting-edge application of this technology to urethral diseases. Furthermore, although certain bioprinting techniques have been previously used to fabricate urethral constructs, there remains a notable gap in the literature concerning the application of advanced printing methods to generate complex, multilayered, tubular structures. In light of this, our review describes current gaps in the utilization of 3D bioprinting technology for the creation of cell-seeded, vascularized, mechanically, and structurally modifiable multilayered structures suitable for clinical translation.

In this regard, the application of 3D printing technologies for the treatment of urethral diseases will be discussed with a focus on the mechanical properties of bioinks and various 3D bioprinting methods. These methods will be briefly compared with traditional techniques used for engineering 3D urethral tissues, such as electrospinning and molding. The focus will be on the anterior urethra, where the external structure and tissue characteristics present a significant challenge for functional penile reconstruction in both congenital and acquired conditions. Finally, ongoing challenges and future directions in urethral tissue reconstruction will be examined, including current limitations for translational applications.

## Methods of 3D bioprinting for urethral tissue construction

3D bioprinting has the potential to engineer synthetic urethral tissues using bioinks, which allow for the development of layer-by-layer structures that imitate the function and architecture of native urethral tissues. Bioinks can be engineered to have tunable mechanical, structural, and biological characteristics that replicate the heterogeneity of the urethral tissue [28]. Additionally, natural or synthetic biomaterials can be combined with primary lower urinary tract cell lines in a spatially patterned deposition, reflecting native differences by anatomic segment [29]. Until recently, a limitation of 3D bioprinting in this regard is that biological materials printed in the air often resulted in poor fidelity. However, the recently developed embedded **freeform reversible embedding of suspended hydrogels (FRESH)** printing approach extrudes bioinks within a yield-stress support bath, ensuring the bioinks remain within the printed configuration until cured [30,31]. Although certain 3D bioprinting methods have already been applied to urethral construct fabrication, others have not yet been used to generate multilayered tubular structures. Box 1 provides a concise overview of the cell-laden 3D printing mechanism and provides a conceptual illustration of the various 3D bioprinting methodologies used in urethral tissue reconstruction.

By selecting a 3D printing method designed to construct multilayer structures, urethral tissue constructs can be designed with distinct regions separated radially to coincide with an inner **urothelial cell (UC)** layer and an external **smooth muscle cell (SMC)**-laden layer (Figure 1) [50–52]. The rationale for this radial partition is twofold, based on both cellular and structural perspectives. SMCs within the vascular corpus spongiosum primarily compose a region of the native urethra that is external to the mucosal and submucosal (lamina propria) layers, while the mucosa contains a continuous, tightly packed multilayered UC structure [53]. Functionally, this arrangement enables the urethra to prevent diffusion of urine and modulate immune control in the urothelium, while spongiosal tissue provides external support for the luminal structure. Bioprinted urethral constructs can facilitate separation between these distinct inner and outer regions [54].

One of the first trials that applied engineered tubular constructs for urethral substitution in a clinical setting was published by Raya-Rivera and colleagues [55]. With a median follow-up of 71 months, this was a small series of five boys with posterior urethral injuries treated with cell-seeded synthetic constructs [55]. In the first stage, autologous cell lines (SMC, UC) were derived from bladder biopsy and seeded onto polyglycolic acid/poly(lactic-co-glycolic acid) (PGA/PLGA) scaffolds. These seeded scaffolds were then used in a tubular urethroplasty to repair 4–5 cm length defects. During the study period, two boys (40%) required a secondary intervention. Despite this, these intermediate results were promising, as all boys were voiding with patent urethras at study conclusion. Likewise, serial urethral biopsies demonstrated urothelial and smooth muscle bilayered architecture. However, a remaining limitation is that the range of phenotypic presentations requires the ability to create reproducible, personalized constructs for wide clinical applicability. In the following sections, recent methods of 3D bioprinting for urethral tissue reconstruction will be discussed.

## Direct bioprinting of urethral tissue

**Figure 2.**
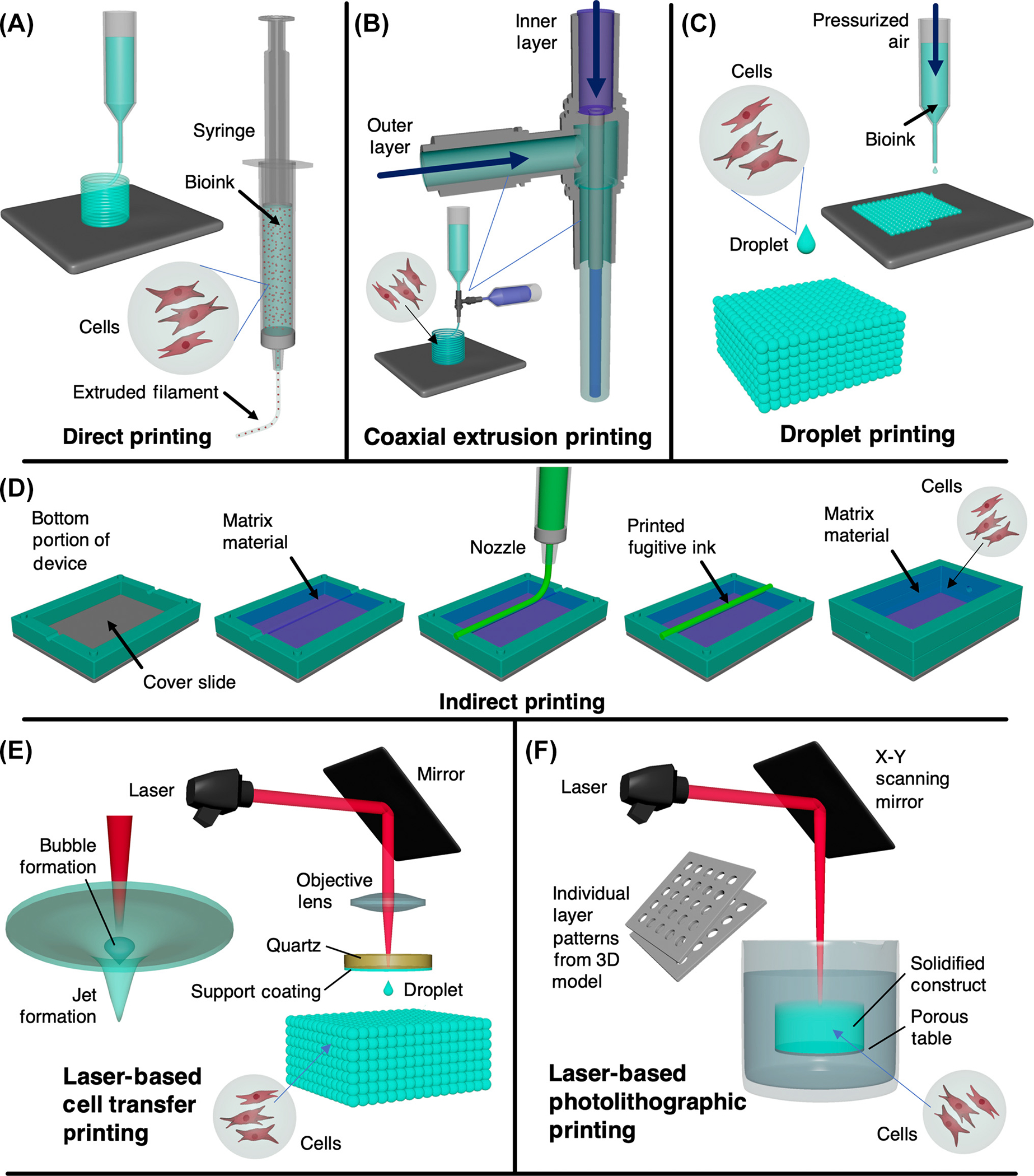
Common 3D bioprinting methods for engineering urethral tissue constructs. (A) Direct inkjet-based bioprinting; (B) coaxial extrusion; (C) droplet-based bioprinting; (D) indirect inkjet-based bioprinting; (E) laser-based forward transfer bioprinting; (F) laser-based photolithographic bioprinting.

**Figure 3.**
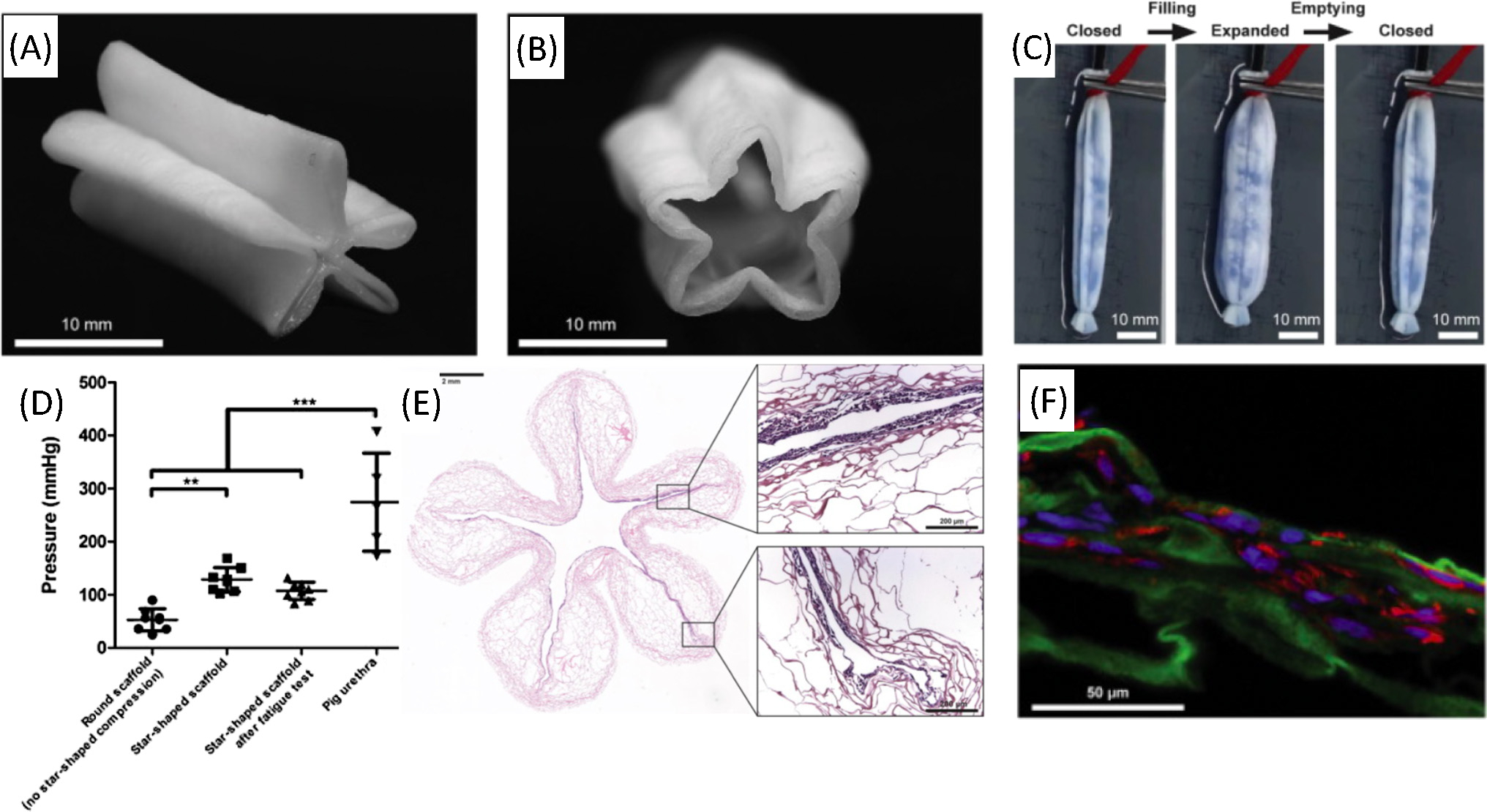
Star-shaped scaffold with radial elasticity for hollow organ regeneration. Macroscopic view of the scaffold in (A) closed and (B) partially open position; (C) expansion and relaxation (folding, unfolding) of the scaffold after injection and removal of water; (D) burst pressure of round scaffolds without star-shaped compression, star-shaped scaffolds before and after fatigue test (both *n* = 8), and of native pig urethras (*n* = 5). Tubes were closed at both ends, and water was pumped into the lumen until rupture while continuously monitoring the pressure. Bars represent the mean ± standard deviation. One-way ANOVA with Bonferroni *post hoc* test, ***P < 0.0001; (E) **hematoxylin and eosin staining (H&E)** staining of cross-sections of cell-seeded star scaffold. (F) Immunostaining of dynamically cultured scaffold stained for cell nuclei with DAPI (blue), type I collagen (green), and cytokeratin 18 (red). Reproduced, with permission, from [57]. Copyright 2017, Elsevier.

Direct bioprinting methods utilize pressurized forces to direct the flow of bioink from the nozzle, creating the shape of printed products without any necessary casting (Figure 2A). Such extrusion methods are simple in experimental execution and can print multicomponent cell-laden constructs [56]. Versteegden and colleagues used direct bioprinting to create a collagen-based construct, devising a star-shaped tube that expanded with luminal flow. The scaffold was seeded with human UCs and cultured in a bioreactor under dynamic conditions mimicking urination (pulse flow of 21 s every 2 h) [57]. The porous tubular collagen scaffold was compressed between two surfaces under a rolling motion resulting in a compressed tubular scaffold (Figure 3A,B). The scaffold was manually compressed around a five-point star-shaped mandrel, fixed with custom-made clamps, and crosslinked in a star-shape position using **1-ethyl-3-(3-dimethylaminopropyl) carbodiimide/N-hydroxysuccinimide (EDC/NHS)**. This unique geometry was selected to mimic the physiological features of the spongy urethra and its radial elasticity. The 3D bioprinted structure provided control over the hydrodynamic pressures exerted on urethral tissue during urine flow by allowing for the expansion of the luminal cross-sectional area when fluid was excreted (Figure 3C). Burst pressure tests of the geometric modification revealed a significant increase in mean burst pressure in star-shaped versus round configurations, with maintenance of mechanical characteristics after exposure to 1000 filling and emptying cycles (Figure 3D). Histologic analysis demonstrated approximately 75% of UC luminal surface coverage after 6 days, with retained cell line-specific markers (Figure 3E,F). Beyond achieving cellular proliferation along the inner construct surface and maintaining its mechanical integrity after cyclic flow, the unique luminal shape maintained radial elasticity that more closely resembled native tissue behavior than previous designs. The outcome of this study suggests that the hydrodynamics of fluid flow must be considered when designing tissue engineered constructs, ensuring that the materials and design facilitate urethral configurations throughout voiding cycle.

In another study, Xu and coworkers introduced a hollow tubular urethra composed of PLGA/poly (ε-caprolactone) (PCL)/triethyl citrate (TEC) (70:30:6) using 3D printed polyvinyl alcohol (PVA) as the sacrificial template [58]. Results revealed that the PCL incorporation improved scaffold toughness, while the incorporation of TEC improved tensile strength and elongation at break. Importantly, significant improvement in the diffusion of oxygen and nutrients was observed, and the prepared scaffolds were customized for specific lesions using cross-sectional imaging, demonstrating a potential path toward improved wound healing and individualized construct development.

A portable, direct ink writing 3D bioprinting pen was also recently utilized to manufacture poly (2-hydroxyethyl methacrylate) (p-HEMA)/alginate hydrogels [59] to repair rabbit urethral defects. Subsequent retrograde urethrograms demonstrated urethral patency without strictures 6 weeks postoperatively. In addition, after repairing with p-HEMA hydrogels, UCs penetrated the defect site from adjacent tissues, creating a layered structure with minimal local inflammatory response. This study confirmed the potential for an engineered hydrogel-based bioink to enhance wound healing.

## Coaxial extrusion bioprinting of urethral tissue

**Figure 4.**
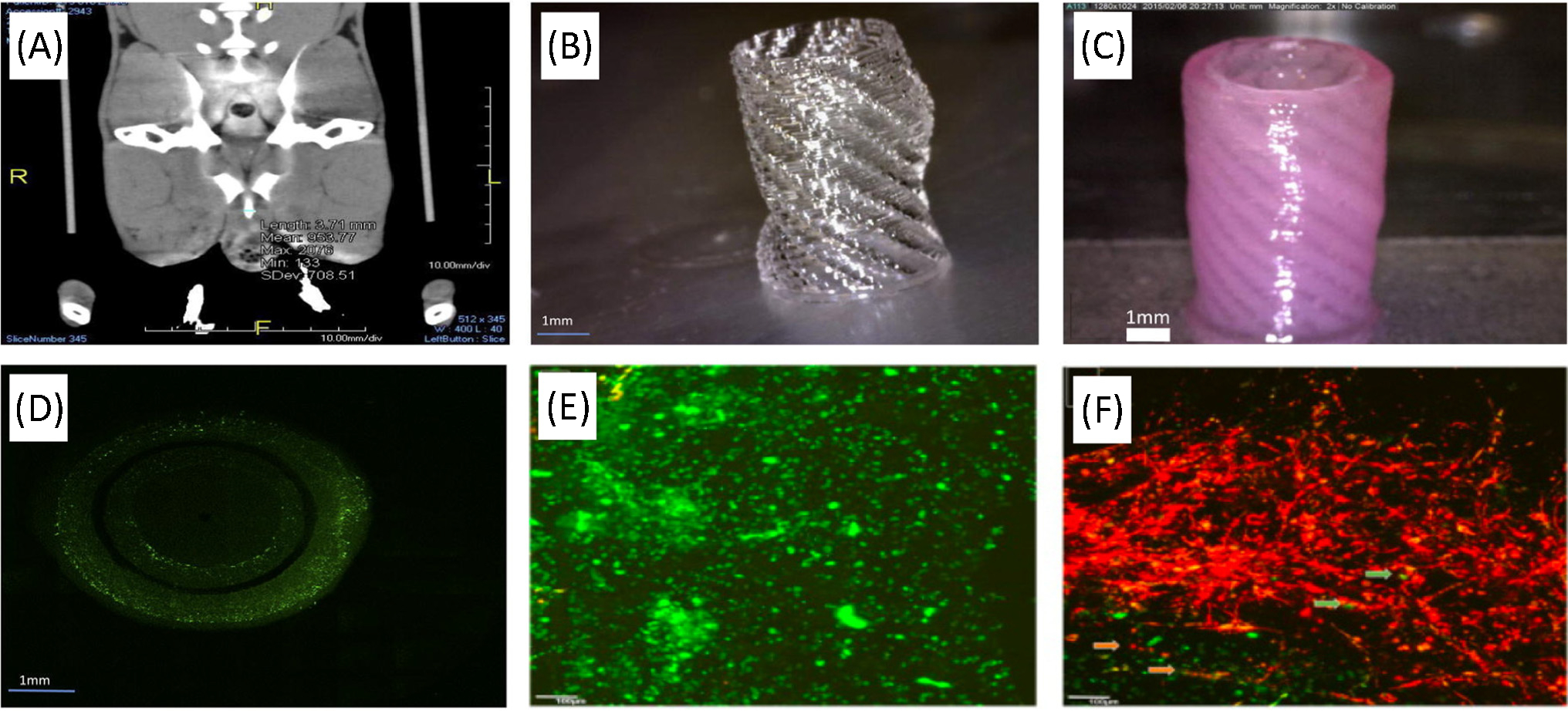
3D bioprinted PCL/PLCL urethral constructs with structural modification to tubularized design. (A) CT scan of a male rabbit urethra. (B) PCL/PLCL (50:50) scaffold with spiral design. (C) Final structure of the bioprinted urethra. (D) The viability and proliferation of UCs and SMCs in the bioprinted urethra. (E) UCs (labeled with PKH67 green fluorescent dye) as seen in the hydrogel component of the bioprinted urethral construct after 7 days of culture. (F) SMCs (labeled with PKH26 red fluorescent dye) in the hydrogel component of the bioprinted urethral construct after 7 days of cell culture. Reproduced, with permission, from [50]. Copyright 2017, Elsevier. Abbreviations: PCL, poly(ε-caprolactone); PLCL, poly(lactide-co-caprolactone); SMC, smooth muscle cell; UC, urothelial cell.

Most recent studies that have applied 3D bioprinting to produce cellularized tubular urethras have utilized coaxial nozzle printers due to their ability to produce multilayered architectures in a single step (Figure 2B). For instance, Zhang and colleagues produced tubular PCL/poly(lactide-co-caprolactone) (PLCL) scaffold mimicking the structural and mechanical properties of urethral tissue through layer-by-layer deposition. This 3D bioprinting process stacked 2D patterns of thermoplastic polymers and cell-laden bioinks [50]. The precision of this method was demonstrated by creating a variation on a classical tubular geometry through the incorporation of a helical ‘ribbing.’ This modification enhanced stretchability while columnar designs improved tensile strength as compared with a simple tubular design. (Figure 4A,B). After printing, the construct was chemically crosslinked (Figure 4C,D) and seeded with UC and SMC cell lines. Initially, the robust PCL backbone of the structure could not support cellular proliferation; therefore, fibrin hydrogel was incorporated within the printed structure to improve cell proliferation. Subsequently, UCs and SMCs maintained more than 80% viability up to 7 days after printing. Both cell types also showed increased proliferation and maintained cell line-specific phenotypes (Figure 4E,F). While this study demonstrated successful *in vitro* formation of cell-laden constructs, the resultant structures were not applied in an animal model.

**Figure 5.**
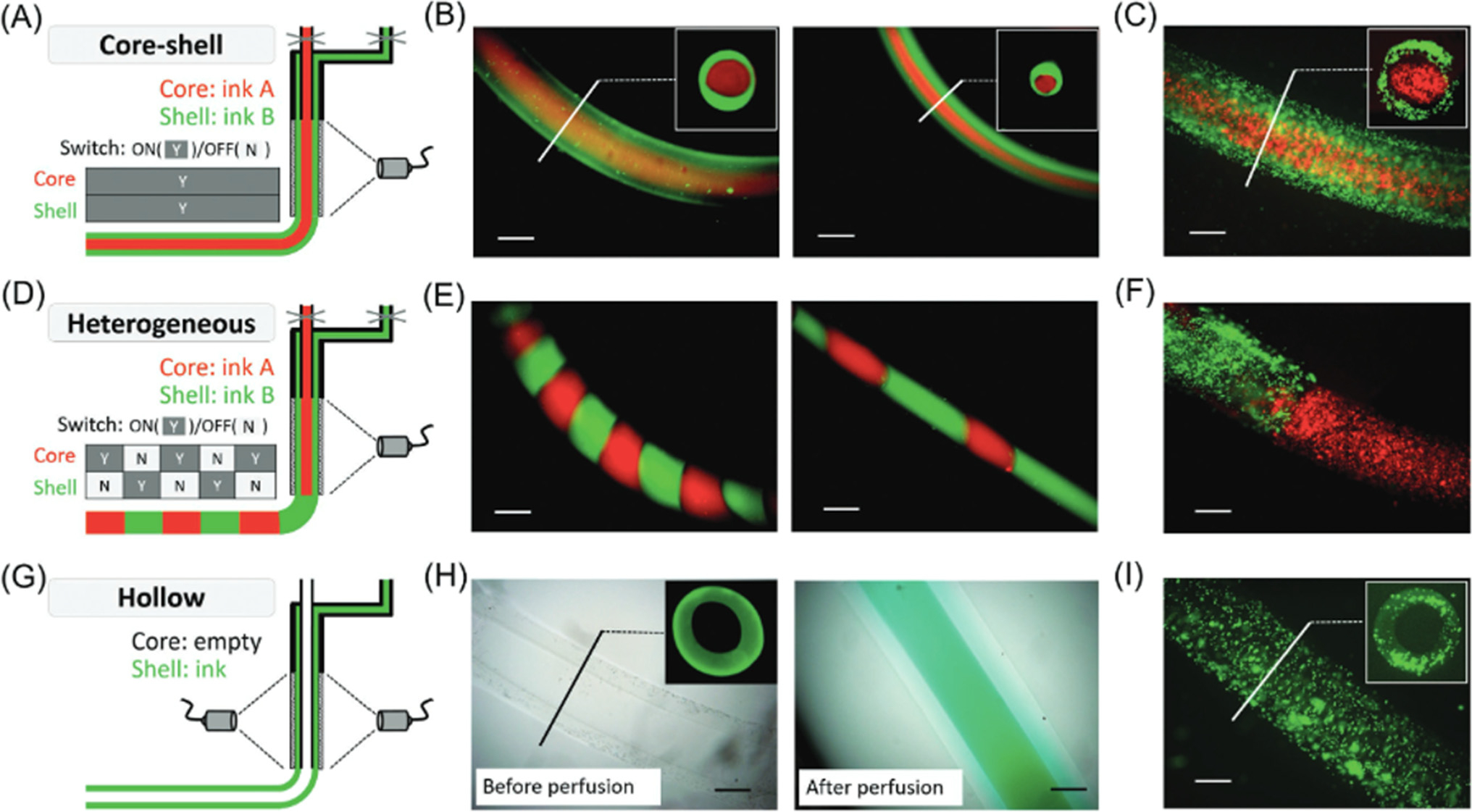
Complex 3D printed structures with *in situ* crosslinking approach. (A) Schematic and representative fluorescence images for printing filaments with core-shell structure using (B) two inks labeled with different fluorophores or (C) two inks containing cells labeled with different dyes. (D) Schematic and representative fluorescence images for printing heterogeneous filaments with intermittent structures using two inks labeled with (E) different fluorophores or (F) two inks containing cells labeled with different dyes. (G) Schematic for printing hollow filaments using a longer core coaxial nozzle and representative images of printed hollow tubes (H) either before or after perfusion with a dye solution or (I) with cells in the printed tubes. Scale bars are 500 μm. Reproduced, with permission, from [52]. Copyright 2017, Wiley.

Ouyang and colleagues similarly utilized a coaxial extrusion technique and a bioink based on the **gelatin methacryloyl (GelMA)** and poly (ethylene glycol) diacrylate (PEGDA) in combination with modified hyaluronic acid (HA). The applied extrusion procedure followed by an *in situ* crosslinking strategy created multilayer and heterogeneous cylindrical structures with high cellular viability (>90%) (Figure 5) [52]. By controlling the on/off status of core and shell channels (Figure 5A–C), heterogeneous filaments were printed with a programmable distribution of multiple inks or cell types along their length (Figure 5D–F). In addition, hollow tubes were printed using a longer core needle to allow for irradiation of the shell before the introduction of core material (Figure 5G), forming hollow structure (cell-laden) filaments (Figure 5G–I) that could be perfused.

In another study, Pi and coworkers designed a multichannel coaxial extrusion system using a blend of GelMA, eight-arm poly(ethylene glycol) (PEGOA), and alginate capable of developing long segments of tubular constructs with controlled layer deposition. Constructs with an inner diameter of 663 μm and outer diameter of 977 μm were printed in a single step [51]. Cells seeded onto the inner layer (human UC) and outer layer (human SMC) demonstrated high levels of viability after 7–14 days of coculture, while distinct layered boundaries were maintained. Subsequently, the expression of cell line-specific UC and SMC markers was observed.

Put together, various studies have demonstrated that coaxial extrusion methods can be used to achieve scaffolds with improved target architectures and optimal mechanical parameters supporting cellular growth postprinting. However, knowledge regarding their translational potential is limited as *in vivo* evaluations of these printed structures have not yet been completed.

## Droplet bioprinting of urethral tissue

Droplet-based bioprinting (DBB) methods are desirable due to their ability to replicate the details of a 3D scan with high fidelity [60]. Droplet-based techniques precisely deposit bioink to form printed structures via a noncontact extrusion tip (Figure 2C). Three different techniques have emerged for DBB according to the method of extrusion, including thermal, electrostatic, and piezoelectric drop on demand [60]. In thermal DBB, a current generated to a heating medium over precise timescales allows for control over the formation of droplets, the size and rate of which can be tuned in printing. With the piezoelectric-based approach, the current is driven to a piezoelectric actuator, which generates mechanical stress and induces the locomotion of a small volume of bioink which coalesces into droplets. Electrostatic bioprinting, in contrast, ejects droplets using an electrostatic force. Inkjet-based droplet printing pressurizes the bioink suspension at the nozzle tip. Cell survival in DBB can be affected by disruption to cell membranes or heat spikes caused by repeated droplet formation, which may affect the cellular proliferation of the printed construct. However, the highly precise degree of control that DBB offers may provide new avenues to improve the efficacy of urethral constructs and create highly personalized scaffolds for surgical use.

While not yet utilized in the context of urethral tissue, DBB allows for the fabrication of constructs with precise dimensions and definitions, suggesting its potential for urethral tissue engineering applications [60]. At present, previously described attempts to create 3D bioprinted urethral scaffolds have focused solely on a UC-laden inner and SMC-laden outer layer. However, transitional urothelium differentiates in a layered fashion with umbrella, intermediate, and basal cells, composing a stratum of individually functional cells rather than a monolayer [53]. While it is challenging to reflect this detail in 3D printed layers with coaxial extrusion or direct bioprinting, the capacity to recreate such histologic detail could improve the regenerative capabilities of replacement constructs. The droplet-by-droplet precision of DBB may be the key to engineering urethral scaffolds with high resolution.

## Indirect bioprinting of urethral tissue

Indirect extrusion bioprinting is another method of bioprinting in which the bulk hydrogel contains a considerable quantity of sacrificial material which is thermally or chemically removed postprinting (Figure 2F). This technique shares some similarities with the direct FRESH approach, which also involves using a support matrix. However, in indirect extrusion bioprinting, the process is distinct in that it prints the desired ink around a fugitive ink, which serves as a temporary support material to create the scaffold structure. The final form of the scaffold is achieved by separating or dissolving the fugitive ink. Indirect approaches have yet to be applied to designing interventions for the urethra, though they have been used for the fabrication of vascular tissues. For example, Lee and colleagues developed a vascular network by using an indirect bioprinting technique and sacrificial gelatin to frame hollow collagen fibers [61]. The method showed great potential in 3D bioprinting for vascularized tissue fabrication, creating vascular channels while printing cells and matrix in desired 3D patterns, and serving as an experimental tool for studying vascular remodeling and maturation under 3D flow conditions.

As urethral tissue engineering approaches continue to develop, indirect bioprinting has a distinct potential to realize constructs with biomimetic physiological properties. Indirect bioprinting advantageously uses a sacrificial material to support softer materials in printing. Using soft and elastic biomaterials such as elastin-like polypeptides may serve in the fabrication of constructs that more closely mimic the elasticity of native urethral tissue. A limitation may be that the soft nature of these materials can affect the feasibility of the resulting construct’s printing, handling, and suturing. Due to this, it is anticipated that the use of hybrid materials or crosslinking will be required.

## Laser-based bioprinting of urethral tissue

Another advanced printing method that promises high control and resolution is laser-based bioprinting (LBB) (Figure 2E). This method relies upon the use of a laser beam that photo-stimulates the interface between an energy-absorbing intermediate, such as gold, or titanium, and the bioink, which contains a sacrificial material. This sacrificial ‘donor layer’ is vaporized under laser stimulation, which generates a high gas pressure propelling the bioink compound toward the printing surface [62]. LBB is generally characterized by its high printing resolution, slower fabrication speed, and orifice-free droplet-based nature which is less susceptible to clogging-related failures associated with highly viscous bioinks [63]. Xiong and coworkers have shown the effectiveness of utilizing LBB for engineering tubular and bifurcated constructs, using an alginate-based bioink to produce layer-by-layer architectures in suspension which can subsequently undergo chemical crosslinking [64]. This study demonstrated that the printing resolution could be improved by adjusting the vertical step size when printing. The author presented this variation to tune the density of the tubular construct, a property that has significant histologic implications. Though yet to be applied to the urethra, LBB may provide another path to create constructs with stratified cell layers. LBB likewise avoids the challenges in printing that may occur with modifications in hydrogel viscosity that arise in DBB. However, it will be necessary with LBB to consider restrictions imposed by thermal stress caused by laser irradiation, which can alter thermosensitive hydrogel architectures and bioinks during printing.

## Biomaterials used for engineering urethral tissue constructs

Since key properties of an ideal biomaterial in urethral replacement include favorable biocompatibility, target biodegradability, and elastic tensile properties [65], the design of a biomaterial platform that can address these aspects is a high priority [66]. As can be concluded from the previously described microfabrication techniques and relevant studies to date, wide ranges of natural and synthetic polymers have been used for the synthesis of 3D urethral scaffolds. Naturally derived materials such as gelatin, alginate, collagen, and silk at times are limited for surgical application due to constructs of inadequate strength for handling and suture placement. Although collagen is a primary **extracellular matrix (ECM)** component, only one report has demonstrated the direct printing of carbodiimide crosslinked collagen for ureteral or urethral reconstruction; this limited availability may be due to inadequate mechanical properties and slow gelation rate (burst pressure ~ 132 ± 22 mmHg) in its unmodified state [57]. More extensively explored, GelMA composites have been 3D bioprinted for multilayer preparation of urethral tubes which may be due to their biocompatibility, accessibility, and ease of crosslinking [51]. Silk fibroin has also garnered attention in the context of 3D printing or electrospinning due to its exceptional mechanical properties; while this material generally suffers from low printability, combining it with other materials can provide increased mechanical strength [67]. Finally, two strategies can be utilized when considering biomaterials with weak printability like alginate. First, a supporting bath filled with a crosslinking solution can be used during the extrusion printing process, in which gelation occurs upon injection into the bath [68]. Alternatively, dual syringe applicators can be operated as a tackle, where polymer solutions and gelators are held in separate syringe reservoirs and meet at the nozzle for the gelation [51].

By contrast, synthetic polymers such as PCL, PVA, polyethylene glycol (PEG), HEMA, PLGA, PLCL, poly(lactic acid) (PLA), and poly(L-lactic acid) (PLLA) are broadly characterized by their greater tensile strength relative to biopolymers. As such, these materials may be more suitable for surgical application, enhancing suturability and operative handling. As noted previously, a synthesized urethral construct has been created using PCL, an FDA-approved synthetic polymer, but has not been applied in an *in vivo* environment. PLA, which undergoes degradation into lactic acid or to carbon dioxide and water after contact with biologic media, has also undergone investigation for urethral construction through molding; nevertheless, it demonstrated poor thermal stability and low crystallinity [69]. Another polymer of interest is PLGA, which has been explored in the context of urethral construction and has demonstrated adhesive properties [58].

In conclusion, while various materials have been utilized for creating 3D urethral tissue constructs, current challenges involve the selection of bioinks with optimal physical properties to achieve functional cell-laden constructs which can then be evaluated effectively in relevant animal models. Therefore, advancements in materials design for urethral tissue reconstruction are focused on the development of hybrid constructs by combining naturally derived and synthetic materials. Additionally, the incorporation of more elastic biomaterials, such as elastin-like polypeptides and tropoelastin, shows promise in overcoming these shortcomings. Furthermore, the inclusion of ECM within synthetic scaffolds offers opportunities for enhancing the biological properties suitable for UC and SMC proliferation.

## Microfabrication of urethral tissue constructs beyond bioprinting

Advancements in urethral tissue fabrication have not been limited to bioprinting, as techniques such as electrospinning and molding have also been explored. For example, electrospun urethral scaffolds have been tested *in vitro* and *in vivo*, showing successful function restoration following urethroplasty (Table 1). In one study, Hu and colleagues applied PLGA/gelatin constructs for tubularized urethral replacement in a canine model [70]. Although all animals voided spontaneously in the early postoperative period, varying degrees of urethral strictures developed after 30 days. This study highlights the challenges of achieving wound healing and vascularization in lengthy urethral repairs. While electrospun scaffolds show promise for concurrent cell implantation and delivery of bioactive agents to improve local vascularization and wound healing, their development is still in the early stages.

By contrast, molding-based techniques necessitate the generation of a mold of the negative space within the lumen while 3D bioprinting allows for the direct reconstruction of a damaged urethral region by replicating the topographical contour of a scanned image [71,72]. If personalization is necessary, the mold is used to shape the tissue accordingly. Consequently, 3D bioprinting is seen as a method capable of producing structures resembling molded constructs, but with fewer and simpler steps.

## Concluding remarks and future perspectives

The high risk of postoperative complications such as fistula, diverticulum, or stricture following urethroplasty, is associated with abnormal urine flow patterns arising from the mismatched structure and tensile properties of replacement tissue and are specific to the urethral segment being addressed. These complications highlight the need for customizable tissue constructs with precise mechanical and structural properties. Current tissue repair options are incapable of sufficiently replacing the urethra and its endogenous characteristics and functions. To address this challenge, 3D bioprinting has emerged as a promising technology to generate scaffolds with target structural, mechanical, and biological properties [54].

Among bioprinting techniques, extrusion-based bioprinting has been the primary method evaluated for urethral application to date. Extrusion-based bioprinting has distinct advantages due to its ability to produce multilayer architectures in a single step and create tissue heterogeneity using syringe head interchangeability and positioning of the printing axis [56]. However, *in vivo* investigations are required to evaluate its translational applicability. DBB, on the other hand, offers superior replication of 3D scans with higher fidelity and resolution, but it can impact cell survival through membrane disruption or heat spikes [60]. Meanwhile, LBB method provides high resolution and improves feasibility of printing viscous biomaterials but has slower fabrication speeds [63]. LBB may also face limitations in urethral applications due to thermal stress generated during laser irradiation, altering thermosensitive hydrogel architectures and bioinks. The use of DBB, LBB, and indirect bioprinting has yet to be reported for urethral application, necessitating initial *in vitro* evaluation. Assessing their ability to precisely pattern tissues at single-cell resolution will be needed in the development of complex urethral structures.

Beyond 3D bioprinting, mold-based methods have been historically used independently or as an adjunct modality. While mold-based methods require longer processing times due to simultaneous shape formation and cell seeding, bioprinting directly translates scanned images into customized dimensions. Additionally, molding necessitates creating a mold of the empty space within the structure for personalization, whereas bioprinting achieves this directly. Bioprinting also provides greater control over the texture and structure of the constructs, as materials are deposited in a precise manner, resulting in a more efficient and accurate process for generating engineered structures for the urethra. An additional modality discussed, electrospinning, has the potential to confer finely-tuned mechanical properties to materials and to create layered constructs with varied fiber orientation, size, or directionality by layer. However, the electric field imposed during electrospinning limits the incorporation of cells during construct fabrication [76,77]. Although electrospinning enables the creation of complex layered constructs for *in vitro* seeding, it lacks the structural precision and cell seeding inherent in the 3D bioprinting fabrication process.

In the selection of future approaches, the researcher must carefully consider the pros and cons of each technology, focusing on those that best align with translational goals from the start of the study (see Outstanding questions). For instance, when the mechanical performance and radial layered organization of urethral tissue scaffolds are important, electrospinning may offer unique advantages. It allows for the creation of multilayered structures with varying topographical characteristics, and the spinning process itself generates porous constructs that enhance material elasticity. By contrast, 3D bioprinting may be more advantageous when precise control over the macro- and microstructure of a seeded construct is desired, and it offers significant personalization advantages for various conditions.

Ongoing challenges to translation of 3D printing to urethral clinical applications include the biocompatibility of the bioinks, safeguarding against immunogenicity and adverse reactions upon implantation; preservation of cell viability during and postprinting which is influenced by both the printing technique and the choice of bioink; integration of the 3D-printed urethral construct with host tissues; ensuring adequate vascularization, resulting in tissue fibrosis or graft necrosis; functional restoration, including early return to voiding without urinary extravasation in the absence of prolonged **urethral catheterization**; innervation of urethral constructs, with appropriate biochemical cues enhanced by urothelial organization consistent with urethral segment; customization of constructs to accommodate patient-specific anatomic variations; and long-term durability that remains functional and exhibits normal growth and aging patterns across the lifespan that mimic adjacent tissue.

To advance 3D bioprinting for clinical translation of urethral tissue reconstruction, further research is needed to improve structural design, bioink compositions, mechanical properties, degradation profiles, cell viability, construct sterility, and manufacturing processes. Additionally, adapting bioinks to guide biological processes like wound healing and vascularization *in vivo* is vital for successful urethral regeneration in challenging environments. Strategies involving seeding with progenitor cells [20], addition of nanoparticles or bioactive factors [19] can be explored for targeted immunomodulation, thereby enhancing vascularization and innervation of the implanted construct. 3D bioprinting has the potential to leverage these bioactive components while achieving personalized topography, mechanics, and structure for urethroplasty, transforming urethral condition treatment for all age groups. By providing customizable constructs that reduce complications without needing tissue grafting, 3D bioprinting can surpass the limitations of standard tissue replacement methods for both children and adults affected by urethral disease.

Finally, beyond achieving the ultimate scaffold, ethical and legal considerations remain pressing concerns in this emerging field. Current urethroplasty outcomes are limited not only by the graft materials used but also by an extensive learning curve that affects surgical outcomes [78]. It is vital that any technological advancements in this field aim to decrease current care inequities by ensuring that the solution can be consistently and effectively applied. Furthermore, cost considerations will affect the broader accessibility of this innovative treatment modality. Ongoing research endeavors must therefore foster advancements in 3D printing and material selection that are reproducible, cost-effective, and will enhance clinical outcomes for patients affected by urethral disease across care settings.

## Figures and Tables

**Table 1.** Microfabrication of 3D urethra tissue constructs^a^

Material	Method	Key results	*In vitro/in vivo* experiments	Refs
Cell-laden PCL/PLCL blend	Direct bioprinting	•Multilayered cell constructs •Cell viability achieved with fibrin insertion •Biomimetic mechanical properties	*In vitro*; NZW Rabbit bladder UCs and SMCs	[50]
PVA cryogel/ PLA mold	Fused deposition modeling	•Geometric, mechanical, and dynamic mimicry of urethra •Use of thermoplastic polyester biomaterial	N/A	[69]
GelMA/alginate/PEGOA	Coaxial extrusion	•Multilayered cell constructs •Single-step fabrication •Tunable layer printing	*In vitro*; human bladder SMCs and UCs	[51]
PLA copolymer scaffold	Solvent casting/particulate leaching	•Stable degradation profile •High porosity with interconnected network •Appropriate cell viability	*In vitro*; adult dermal fibroblasts	[73]
Collagen	Direct bioprinting	•Radial elasticity grants greater fatigue endurance for scaffold	*In vitro*; **SCaBER** cells	[57]
PLGA/PCL/TEC	Direct printing	•Customized for specific lesions with medical image data • >90% cell proliferation	*In vitro*; L929 fibroblast cells	[58]
p-HEMA/sodium alginate	Portable direct ink writing 3D printing pen	•Biocompatible •Unobstructed urethra without evident stricture at 6-week endpoint	*In vivo*; L929 cells; *in vivo*; NZW adult rabbit	[59]
PCL/silk fibroin/collagen	Electrospinning	•Oral mucosal **EpiCs** growth •Interconnected porous network and uniform structure	*In vitro*; oral mucosal EpiCs	[67]
PLGA, PLGA/gelatin	Electrospinning	•Regeneration ofcellular networks near scaffold tips but insufficient in interior •Varying degrees of urethral strictures in the reconstructed urethras	*In vitro*; human UCs *In vivo* canine model	[70]
PLLA/gelatin	Electrospinning	•Upregulated phenotypic expression of UCs and SMC • Urethral patency and reconstructive modeling *in vivo*	*In vivo*; NZW rabbit model	[74]
PLLA/PEG	Electrospinning	•Biocompatible •No urethral stricture or urinary fistula *in vivo* •Urethral epithelium regeneration	*In vivo*; NZW rabbits, scaffolds with hAMSCs	[75]
PLA/PLCL	Molding	•Mechanical stretchability (300 MPa-week 8/137.9 MPa-week 10) higher than sheep bladder (0.45 ± 0.12 MPa) • Sufficient degradation profile (6–8 weeks)	*In vitro*; human UCs	[71]
Silicone	Molding	•Long-term healing of mesothelial cell-seeded compounds *in vivo* •Presence of layered urothelium and SMCs in the seeded cohort across endpoints (1,2, and 6 months postoperatively)	*In vitro*; mesothelial cells *In vivo*; NZW adult rabbit	[72]

## Highlights

Urethral disease profoundly affects the quality of life and healthcare costs, necessitating better tissue replacement and repair approaches due to high complication rates in complex repairs.

Current tissue sources lack healthy urethral structural and mechanical properties, but 3D bioprinting resolves this by overcoming tissue scarcity and replicating the complex structural properties of the urethra.

3D bioprinting can revolutionize personalized urethral repairs, adapting to individual anatomical variations and disease traits with customizable physical properties.

Most bioprinting studies have concentrated on *in vitro* evaluation, showing the potential to culture lower urinary tract cell lines with hybrid and biologic materials.

Key bioprinting advancements, including bioink modifications and the addition of bioactive factors, are vital for the development of urethral regeneration.

## Box 1. Principles and methods of cell-laden 3D bioprinting

3D bioprinted scaffolds with live cells, referred to as cell-laden constructs, offer a transformative approach to addressing three key challenges in urethral tissue engineering: the structural and mechanical mismatch between native and reconstructed urethra, the absence of individualized repair solutions, and inadequate wound healing and vascularization. This advanced technology allows for the precise delivery of specific cells by location, resulting in constructs that better replicate the structural and cellular distribution of native organs [24]. This process involves three distinct stages: pre-bioprinting, bioprinting, and post-bioprinting [32]. In pre-bioprinting, a 3D design of the desired structure is created using cross-sectional imaging (e.g., computational tomography or MRI) [33] and/or surface imaging (e.g., photogrammetry, structured light scanning) [34] to define the unique anatomic defect. Concurrently, cells isolated from autologous native tissue are expanded and mixed with a hydrogel precursor to serve as a supportive matrix. Additional nutrients or sacrificial materials are added based on the bioprinting method chosen. The cell-laden bioink is then printed to create a 3D construct using the selected bioprinting technique. In the post-bioprinting phase, the printed structures are cultured in conditions supportive of cellular proliferation and growth [35]. Moreover, ECM proteins from the seeded cells may partially or completely replace the scaffold during this phase [36].

Hydrogel-based bioinks, which are typically preferred, allow for facile variation in construct geometry followed by crosslinking for enhanced stability and improved mechanical properties [37]. These bioinks can be naturally derived or synthetic polymers. Natural bioinks have innate bioactivity due to naturally occurring peptide sequences or conformational motifs that enhance cell and tissue integration and biocompatibility [38]. By contrast, synthetic bioinks offer more control over molecular weights and crosslinking densities, allowing for precise control of specific mechanical properties such as elastic modulus and shear stresses [39]. Hybrid mixtures of natural and synthetic polymers have also been increasingly used during the past decade as advanced composite bioinks [40,41].

The vast array of hydrogel and protein-based bioinks provides the opportunity to select materials meant to recreate specific mechanical properties of healthy endogenous urethral tissue [42]. In addition, the use of different bioinks and cells that can be tailored by layer allows for the creation of optimized microenvironments for each cell type. This aims to reproduce an organ-specific ECM-like microenvironment, regenerating a construct resembling adjacent healthy tissue.

A conceptual representation of various 3D bioprinting techniques for the reconstruction of urethral tissues is presented in Figure 2 which includes direct printing, coaxial extrusion printing, droplet printing, indirect printing, and laser-based printing. Each 3D bioprinting method illustrated in Figure 2 allows for the precise placement of cells and materials in a layer-by-layer fashion. This level of precision enables the creation of complex, patient-specific structures that closely match the unique anatomical features of each patient’s urethra. By tailoring the architecture and mechanical properties of the 3D printed construct to mimic those of the native tissue, the issue of structural and mechanical mismatch can be mitigated. In addition, traditional methods often use one-size-fits-all approaches, which can be inadequate for patients with unique anatomical variations or specific needs. These 3D bioprinting methods leverage patient-specific data obtained through cross-sectional imaging to create individualized solutions [43,44]. This ensures that the repair is precisely tailored to the patient’s anatomy, reducing the likelihood of complications and optimizing outcomes. Moreover, diverse 3D bioprinting methods can replicate the native tissue microenvironment by integrating the patient cells and providing spatial control over cell placement [45]. This, in turn, can enhance wound healing and vascularization by creating a more suitable environment for cell growth and tissue integration [46–48]. Additionally, the use of hydrogel-based bioinks can offer a scaffold with properties that promote healing and integration, such as providing mechanical support and facilitating nutrient and oxygen exchange [49].

## Outstanding questions

What are the relative efficacy and limitations of different bioprinting techniques (extrusion-based, droplet-based, etc.) for urethral tissue engineering?

Which specific 3D bioprinting techniques can be applied to fabricate urethral constructs with enhanced structure, mechanics, and control over the microenvironment by cell line?

Which biomimetic materials will create structures that have the remarkable elasticity of the urethra, enhance cell proliferation, and remain suturable while limiting urine extravasation?

How can bioinks and growth factors be optimized to promote wound healing, immunomodulation, and tissue regeneration, regardless of urethral disease that often creates tissue fibrosis and limited vascularization potential?

Can ECM-based strategies for urethroplasty be improved to address the issues of high tensile strength and lack of elasticity seen in current commercially available products?

Are there opportunities to combine different fabrication techniques, such as bioprinting and molding, to leverage their respective advantages and create optimal urethral constructs?

How can individualized constructs be consistently developed in conditions that are often not preoperatively imaged using cross-sectional or external imaging modalities?

How can computational modeling and simulation be integrated into the design and fabrication of urethral constructs to optimize both early performance and long-term patient outcomes?

How can the translational gap between animal studies and human trials be addressed, particularly when long and complex urethral defects are repaired?

## Glossary

Buccal: superficial inner cheek graft involves taking a thin layer of epithelialized tissue from the inside of the cheek, used as a tissue alternative in reconstructive surgery.
Corpus spongiosum: the fibromuscular layer that surrounds the penile urethra.
Diverticulum: an abnormal pouch-like bulge caused by the distension or widening of a tubular structure.
Extracellular matrix (ECM): a protein-based acellular framework supporting tissues, organs, and the microenvironment where cells reside, proliferate, or modify their phenotype.
1-ethyl-3-(3-dimethylaminopropyl) carbodiimide/N-hydroxysuccinimide (EDC/NHS): a chemical coupling agent used to link biomolecules for various applications including protein labeling, antibody modification, surface functionalization, and drug delivery.
Epithelial cells (EpiC): form protective layers on body surfaces and inner organs, playing essential roles in exchange, absorption, and structural support.
Fistula: an abnormal connection between a hollow or tubular organ and the body surface, or between two organs.
Freeform reversible embedding of suspended hydrogels (FRESH): a 3D bioprinting method for creating complex tissue structures using a supportive gel to temporarily hold soft hydrogel-containing cells, enabling high-resolution printing and maintaining cell viability.
GelMA: gelatin functionalized with methacryloyl motifs.
Hematoxylin and eosin staining (H&E): a widely used histological technique that colors tissue structures for better microscopic analysis. Hematoxylin highlights nuclei in blue, and eosin colors cytoplasm and other structures in pink or red.
Prepuce: a preputial graft involves taking a piece of skin from the foreskin, which is the fold of skin that covers the tip of the penis, for grafting purposes in reconstructive surgeries.
SCaBER: squamous cell carcinoma of the bladder-derived cell line.
Smooth muscle cells (SMCs): assist in active expulsion of urine through peristalsis (cyclic contraction and relaxation), resulting in an antegrade fluid wave.
Tumescence: penile erections.
Urethroplasty: surgical repair or replacement of the urethra to restore proper urinary function when it is damaged or obstructed.
Urethral catheterization: the process of diverting urine via a tube inserted in the urethra that extends to the urinary bladder.
Urethral stricture disease: narrowing of the urethra is a condition managed by urethroplasty in adults and may develop due to traumatic injury, infection, or malignancy.
Urothelial cells (UCs): specialized cells that cover the inside surface of the urinary tract including the bladder, ureters, and urethra.
